# Successful treatment of corrosive esophageal atresia in a child using combined antegrade-retrograde dilation: a case report

**DOI:** 10.3389/fped.2025.1704549

**Published:** 2026-01-12

**Authors:** Hao Wu, Xiaohan Jiang, Zhiqi Wang, Li Liu, Xuming Mo, Zhining Fan

**Affiliations:** 1 Department of Digestive Endoscopy, Jiangsu Province Hospital and the First Affiliated Hospital with Nanjing Medical University, Nanjing, Jiangsu, China; 2 Department of General Surgery, Jiangsu Province Hospital and the First Affiliated Hospital with Nanjing Medical University, Nanjing, Jiangsu, China; 3 Department of Geriatric Gastroenterology, Jiangsu Province Hospital and the First Affiliated Hospital with Nanjing Medical University, Nanjing, Jiangsu, China; 4 Department of Cardiothoracic Surgery, Nanjing Children’s Hospital, Nanjing, Jiangsu, China

**Keywords:** caustics, child, combined antegrade-retrograde endoscopic dilation, endoscopy, esophageal atresia

## Abstract

Corrosive esophageal injury in children may rarely lead to complete atresia. No standardized treatment exists for pediatric corrosive esophageal atresia. This case demonstrates a novel minimally invasive approach. A 12-year-old male developed complete esophageal atresia after accidental ingestion of industrial alkali. He underwent combined antegrade-retrograde endoscopic dilation (CARD) with balloon dilation and covered stent placement. Oral feeding resumed by postoperative day two, and at three-month follow-up, he reported normal eating without complications. To our knowledge, this represents the first reported use of CARD for pediatric corrosive esophageal atresia, demonstrating it as a feasible and effective minimally invasive alternative to surgery in selected cases.

## Introduction

Corrosive esophageal injury is a serious pediatric emergency, typically caused by accidental ingestion of alkaline agents, which induce liquefactive necrosis and can lead to long-segment strictures or even complete esophageal atresia (1). While endoscopic balloon dilation (EBD) remains standard for strictures, it often fails in long or obliterated segments, necessitating surgical interventions such as gastric pull-up or colonic interposition—procedures associated with considerable risks including anastomotic strictures and reflux (2, 3). The CARD technique, introduced in 2006 and refined thereafter, allows recanalization via simultaneous oral and gastrostomy access under endoscopic/fluoroscopic guidance (4, 5). Although CARD has been effective in adults, its application in pediatric corrosive esophageal atresia has not been previously reported. Here we present the first pediatric case successfully treated with CARD, highlighting its potential as a minimally invasive alternative to conventional surgery.

## Case presentation

A 12-year-old boy was admitted with a history of accidental ingestion of industrial alkali 10 months earlier. Immediately after ingestion, he developed aphonia and was transferred to a local hospital, where he received gastric lavage and nutritional support in the intensive care unit. Despite one week of treatment, he was unable to resume oral feeding, and esophageal stricture was suspected. He was referred to a pediatric hospital in Beijing, where endoscopic dilation was attempted but complicated by perforation. Oral intake remained impossible.

Two months after ingestion, a gastrostomy and jejunal feeding tube were placed. One month later, upper endoscopy revealed obstruction of the proximal esophagus. After six months of home recuperation, repeat endoscopy through the gastrostomy demonstrated distal esophageal obstruction. The patient was then referred to our hospital and admitted with a working diagnosis of post-caustic esophageal stricture.

**Figure 1:**
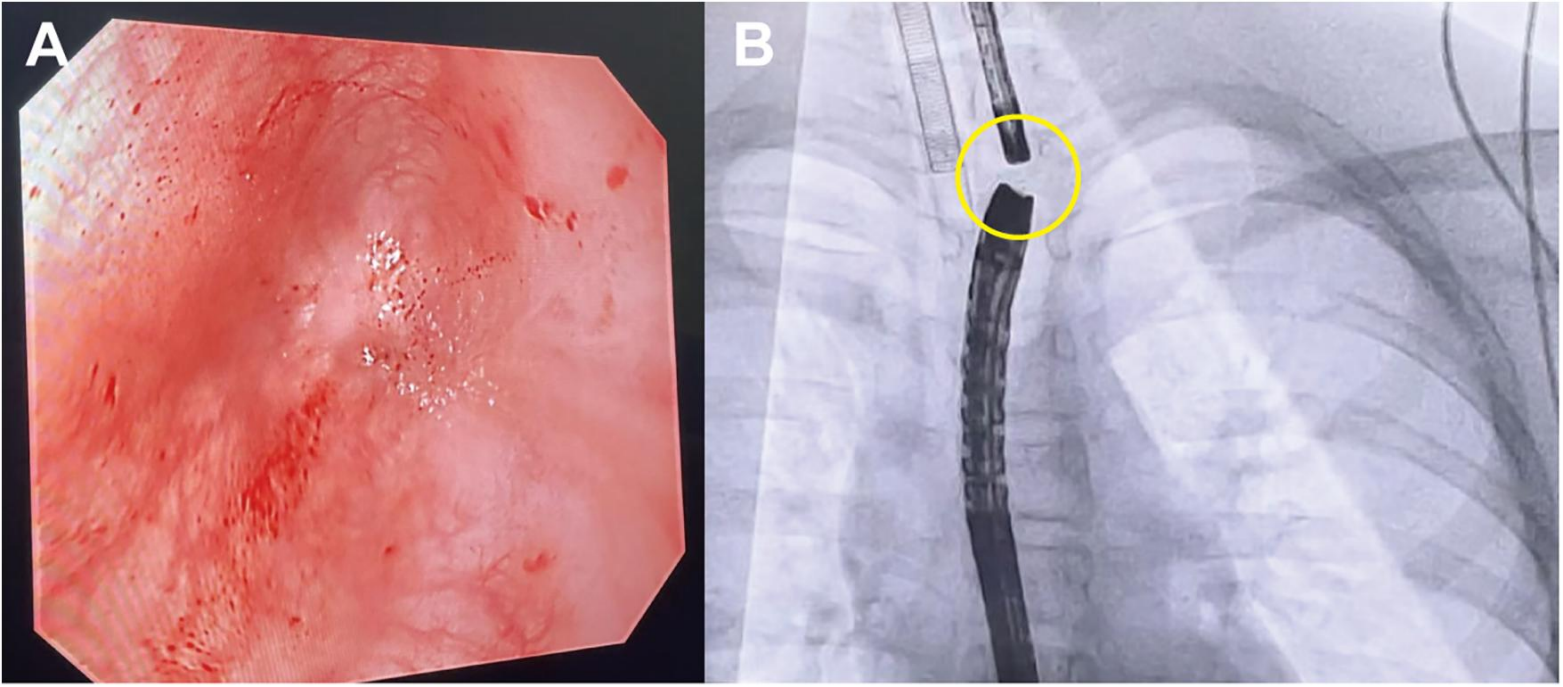
Endoscopic and fluoroscopic confirmation of complete esophageal atresia. Endoscopic image shows complete esophageal atresia with a blind-ending proximal pouch located 22 cm from the incisors **(A)**. The yellow circle indicates the 1 cm distance between the two endoscopes **(B)**.

On examination, he was alert with mild dyspnea. He reported a history of vomiting when attempting to swallow saliva. Breath sounds were coarse but without rales; cardiac rhythm and sounds were normal. A gastrostomy tube and a jejunal feeding tube were in place. Oral endoscopy revealed a blind pouch 22 cm from the incisors (shown in Figure 1A). Endoscopy through the gastrostomy showed a tight cardia stricture, impassable with a standard scope. Contrast fluoroscopy demonstrated a 10-cm stricture above the cardia, with proximal and distal luminal diameters of approximately 3 mm and 5 mm, respectively. Critically, no evidence of an esophageal-bronchial fistula was identified on fluoroscopic imaging or during endoscopic examination of both the proximal and distal pouches. Balloon dilation with a 12-mm catheter was performed, allowing passage of a standard gastroscope. A blind end was again identified 10 cm above the cardia. The preoperative diagnoses were chemical esophagitis with atresia, post-caustic esophageal stricture, and status post-gastrostomy.

**Figure 2:**
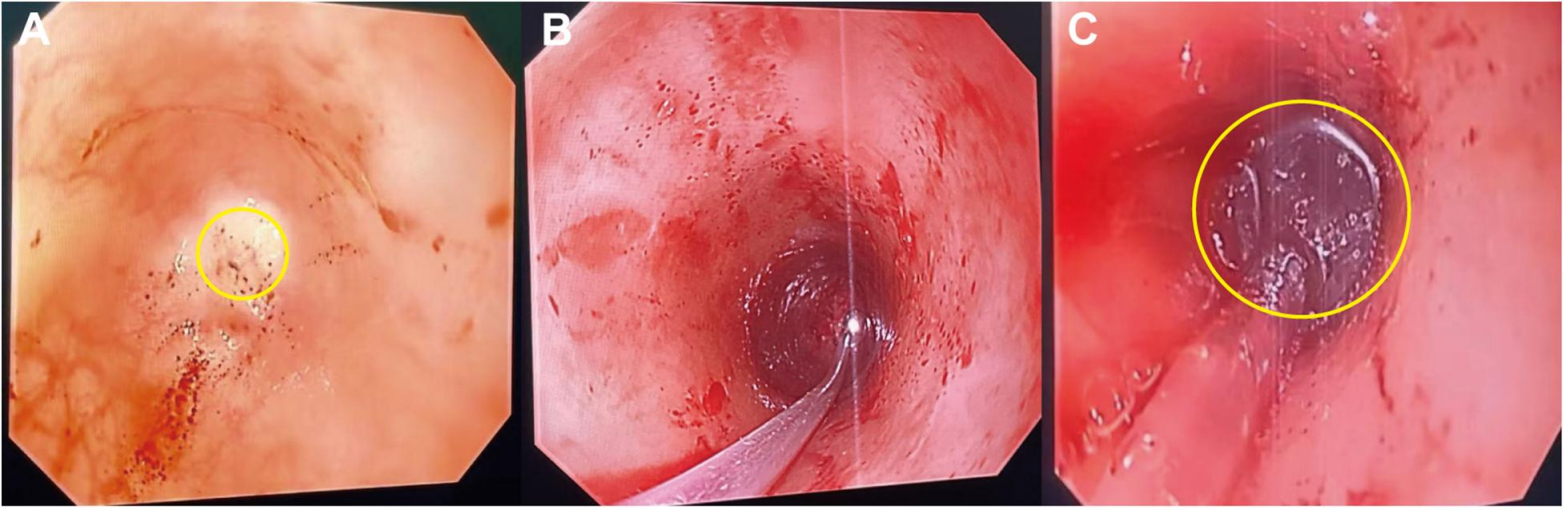
Implementation of CARD procedure. The yellow circle indicates the mucosal puncture needle penetrating the center of the proximal esophageal blind end **(A)**. A guidewire was introduced through the puncture site **(B)**. The yellow circle indicates the dilating balloon **(C)**.

Based on the patient's clinical status, a combined antegrade-retrograde endoscopic dilation procedure was performed. During the operation, gastroscopes were inserted through both the oral cavity and the gastrostomy site until reaching the atretic segment of the esophagus. Fluoroscopy showed the two gastroscopes were approximately 1 cm apart (shown in Figure 1B). Under visualization from the gastrostomy gastroscope, a mucosal puncture needle was inserted via the oral gastroscope to perforate the blind end at its center, successfully penetrating into the distal esophagus (shown in Figure 2A). A guidewire was then placed through the puncture site, followed by sequential dilation using 10 mm and 12 mm balloons to reconstruct the esophageal lumen (shown in Figures 2B,C).

**Figure 3:**
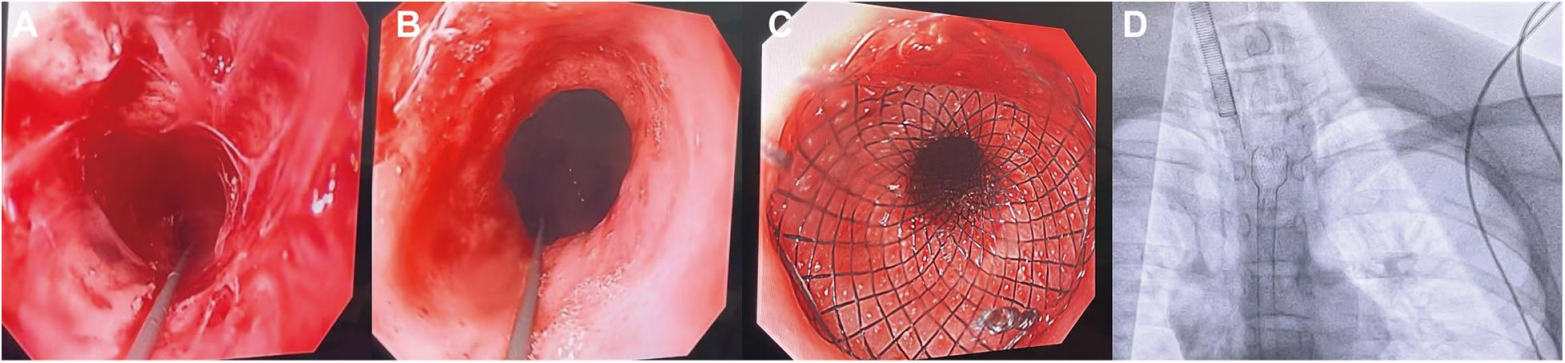
Placement of a fully covered, retrievable esophageal stent. Approximately half of the proximal esophagus shows circumferential stenosis **(A)**. Approximately two-thirds of the distal esophagus shows circumferential stenosis **(B)**. Placement of a 14-cm-long, 16-mm-diameter fully covered, retrievable stent in the esophageal stricture segment **(C)**. Postoperative fluoroscopy confirms the stent is in place **(D)**.

Post-dilation examination revealed a circumferential stricture of about 10 cm in the distal esophagus, with the proximal segment narrowed by approximately 1/2 and the distal segment by about 2/3 (shown in Figures 3A,B). Subsequently, a guidewire was advanced into the stomach via the oral gastroscope, and a covered stent delivery sheath was inserted along the wire, successfully deploying a fully covered retrievable stent measuring 14 cm in length and 16 mm in diameter (shown in Figure 3C). Postoperative fluoroscopy confirmed that the stent was in place (shown in Figure 3D).

The patient underwent the CARD procedure and was able to resume near-normal oral intake by postoperative day two. At three-month follow-up, he reported normal eating without postoperative complications. The patient's parents expressed relief that their child could eat normally again and were grateful that a major surgery was avoided.

He continues to undergo regular endoscopic surveillance. The specific timeline of diagnosis, interventions, and follow-up is shown in Table 1.

**Table 1:** Timeline of clinical events.

Time point	Clinical event
Day 0	A 12-year-old boy accidentally ingested industrial alkali, developed immediate aphonia, and was admitted to the local ICU for gastric lavage and nutritional support.
+1 week	Persistent inability to take food orally; esophageal stricture was suspected.
Referral	At a tertiary pediatric hospital in Beijing, endoscopic dilation was attempted but complicated by perforation; oral feeding remained impossible.
+2 months	Gastrostomy and jejunal feeding tube placement were performed.
+3 months	Upper endoscopy revealed proximal esophageal obstruction.
+8 months	After six months of recuperation, endoscopy via the gastrostomy demonstrated distal esophageal obstruction.
+10 months	The patient was referred to our hospital and admitted with a working diagnosis of post-caustic esophageal stricture.
Admission	Oral endoscopy showed a blind pouch 22 cm from the incisors; endoscopy via gastrostomy revealed cardia stenosis. Contrast fluoroscopy demonstrated a 10-cm stricture above the cardia.
Procedure day	CARD with balloon dilation and placement of a fully covered stent was successfully performed.
Postoperative day 2	Oral feeding was resumed.
3-month follow-up	The patient was tolerating a normal diet without complications; regular endoscopic surveillance was planned.

## Discussion

Corrosive esophageal injury is a severe and potentially life-threatening condition, particularly in the pediatric population, where accidental ingestion of household or industrial alkaline substances remains relatively common. These agents, through their ability to cause liquefactive necrosis, can penetrate deeply into the esophageal wall, leading to extensive tissue damage. In severe cases, this may result in full-thickness injury, long-segment fibrosis, and, albeit rarely, complete esophageal atresia. Managing such injuries in children is complex, as standard endoscopic dilation often proves inadequate. In a cohort study by Radhakrishna et al., approximately 62% of children with corrosive esophageal injury developed strictures, 23% required feeding ostomies, and 12% ultimately underwent esophageal replacement surgery—highlighting the long-term morbidity and the limitations of current therapeutic options (6).

Endoscopic dilation, either with bougies or balloons, has long been the primary approach for managing benign esophageal strictures. However, its efficacy is markedly limited in cases of complete luminal obstruction, where guidewire passage through a fibrotic segment becomes technically impossible. Moreover, repeated dilation procedures are not without risk; esophageal perforation remains a significant concern, and up to 40% of patients continue to experience persistent or recurrent dysphagia despite intervention (7). When endoscopic options fail, surgical reconstruction—such as gastric pull-up or colonic interposition—may be required to restore alimentary continuity. These procedures, while effective in select cases, are highly invasive and carry substantial morbidity, with long-term sequelae including impaired esophageal motility, anastomotic complications, and growth delay.

The CARD technique offers a novel, minimally invasive option for the management of complete esophageal obstruction. Initially described in adult populations, the procedure entails simultaneous endoscopic access via the oral cavity and a gastrostomy site, enabling precise alignment, transillumination, and targeted recanalization of the atretic segment under direct visualization. In a systematic review and meta-analysis, Jayaraj et al. reported a pooled technical success rate of 89% in adults undergoing CARD, with a perforation rate of approximately 6% (8). More recently, a 2024 multicenter case series by Hayat et al. documented successful esophageal recanalization in 86% of patients, sustained improvement in dysphagia in 83%, and a low incidence of adverse events (9).

Despite encouraging outcomes in adult populations, there remains a notable absence of published reports on the use of CARD in pediatric patients with corrosive esophageal atresia. To our knowledge, this is the first case to illustrate the feasibility and clinical efficacy of CARD in a child with complete esophageal obstruction following alkali ingestion. Several anatomical and physiological considerations support the adaptation of this technique in children. The pediatric esophageal wall is thinner and more compliant, potentially enhancing transillumination and facilitating controlled mucosal puncture. Furthermore, preservation of the native esophagus is essential for maintaining normal growth, physiological function, and long-term quality of life—underscoring the appeal of a minimally invasive, organ-sparing approach in this age group.

In this case, precise intraoperative coordination was critical to procedural success. Under fluoroscopic guidance, two gastroscopes were advanced from opposing directions to the proximal and distal ends of the atretic segment. Transillumination confirmed that the blind ends were separated by approximately 1 cm. Using direct endoscopic visualization, a sclerotherapy needle was employed to puncture the fibrotic tissue, allowing for guidewire passage and subsequent serial balloon dilations to re-establish esophageal continuity. Given the presence of a long, circumferential stricture measuring approximately 10 cm, a fully covered self-expandable metal stent (SEMS) was deployed to maintain luminal patency and reduce the risk of early restenosis. SEMS placement has been widely reported as an adjunctive measure in the treatment of refractory benign esophageal strictures. A 2023 review by Sohouli et al. reported clinical success in 68% of pediatric cases, though stent migration occurred in approximately 20% of patients (10).

The postoperative course in this patient was notable for rapid recovery, with oral feeding resumed by the second postoperative day. However, close long-term follow-up is essential, given the risk of complications such as re-stenosis, stent-related injury, and, more rarely, malignant transformation. Survivors of caustic esophageal injury are known to carry a significantly elevated lifetime risk of esophageal squamous cell carcinoma—estimated to be 1,000–3,000 times higher than that of the general population—underscoring the importance of sustained endoscopic surveillance (11). This grave long-term risk, which persists irrespective of the initial management strategy (be it dilation, surgery, or as described in our case, CARD with stenting), is a critical consideration highlighted in contemporary surgical literature as well. Furthermore, as emphasized by Taher et al., the pursuit of organ-preserving procedures is paramount, not only to avoid the morbidity of major surgery but also to facilitate ongoing endoscopic monitoring for early detection of malignancy (12).

While this case highlights the potential of CARD as a feasible and possibly preferable approach for pediatric patients with complete esophageal atresia following caustic ingestion, further investigation is warranted. Prospective multicenter studies and clinical registries are needed to better define optimal patient selection, procedural variables—such as balloon size and dilation pressure—and strategies for post-procedural management. Looking beyond mechanical recanalization, our case underscores a profound and unmet need: the restoration of functional esophageal mucosa. The long-term risk of stenosis and malignancy is intrinsically linked to the impaired regenerative capacity of the tissue after corrosive injury. Therefore, we strongly advocate for a paradigm shift towards foundational research aimed at enhancing esophageal mucosal regeneration. Future efforts should explore innovative strategies in tissue engineering, biomaterial scaffolds, and stem cell therapies to not merely reopen the esophageal conduit, but to truly restore its physiological integrity and resilience. Such breakthroughs are crucial to fundamentally improving the long-term prognosis and quality of life for these young patients.

## Conclusion

This case presents the first reported use of combined antegrade-retrograde endoscopic dilation for pediatric corrosive esophageal atresia. The successful outcome demonstrates that CARD is a viable, minimally invasive, and effective alternative to major surgery in selected pediatric cases.

We recommend further studies and prospective data collection to evaluate CARD's long-term safety, success rate, and indications in pediatric populations. Its implementation may revolutionize the management of complex esophageal lesions in children.

## Data Availability

The original contributions presented in the study are included in the article/Supplementary Material, further inquiries can be directed to the corresponding author.

## References

[1] Kay M Wyllie R. Caustic ingestions in children. Curr Opin Pediatr. (2009) 21(5):651–4. 10.1097/MOP.0b013e32832e2764 19543088

[2] Contini S Scarpignato C. Caustic injury of the upper gastrointestinal tract: a comprehensive review. World J Gastroenterol. (2013) 19(25):3918–30. 10.3748/wjg.v19.i25.3918 23840136 PMC3703178

[3] Contini S Swarray-Deen A Scarpignato C. Oesophageal caustic stricture: still a major clinical challenge. Clin Exp Gastroenterol. (2013) 6:55–64. 10.2147/CEG.S31629

[4] McKaigney CJ Depew WT Bachynski JD. Combined antegrade and retrograde endoscopic recanalization of complete esophageal obstruction. Endoscopy. (2010) 42(4):E132–3. 10.1055/s-0029-124408520405380

[5] Chaves DM Ishioka S Félix VN Sakai P Zilberstein B. Endoscopic management of caustic strictures: an update. World J Gastrointest Endosc. (2013) 5(5):220–9. 10.4253/wjge.v5.i5.220

[6] Radhakrishna V Kumar N Gadgade BD Vasudev RB Alladi A. Sequelae of corrosive injury in children: an observational study. J Indian Assoc Pediatr Surg. (2022) 27(4):435–40. 10.4103/jiaps.jiaps_133_21 36238332 PMC9552654

[7] Adler DG Wang ZJ. Endoscopic therapy for refractory benign esophageal strictures. Pract Gastroenterol. (2024) 48(5):1–9.

[8] Jayaraj M Mohan BP Mashiana H Krishnamoorthi R Adler DG. Safety and efficacy of combined antegrade and retrograde endoscopic dilation for complete esophageal obstruction: a systematic review and meta-analysis. Ann Gastroenterol. (2019) 32(4):361–9. 10.20524/aog.2019.0385 31263358 PMC6595922

[9] Hayat U Khan YI Deivert D Obuch J Altaf A Boger J Combined antegrade and retrograde dilation (CARD) for management of complete esophageal obstruction: multicenter case series. Endosc Int Open. (2024) 12(10):E1199–205. 10.1055/a-2422-8792 39411360 PMC11479796

[10] Sohouli MH Alimadadi H Rohani P Athayde FL Cunha KS Santos HO. Esophageal stents for the management of benign esophageal strictures in children and adolescents: a systematic review. Dysphagia. (2023) 38(3):744–55. 10.1007/s00455-022-10511-8 36038733

[11] StatPearls Publishing. Caustic Ingestions [Internet]. Treasure Island (FL): StatPearls (2023).

[12] Taher H Amgad A Magdi A Magdy A Tantawi HEE Kaddah SN Outcomes of transhiatal colon bypass with or without esophagectomy for establishing continuity after corrosive esophageal burns in pediatric patients. Esophagus. (2025) 22(3):467–74. 10.1007/s10388-025-01114-x 40195178

